# Modern termites inherited the potential of collective construction from their common ancestor

**DOI:** 10.1002/ece3.6381

**Published:** 2020-06-02

**Authors:** Nobuaki Mizumoto, Thomas Bourguignon

**Affiliations:** ^1^ School of Life Sciences Arizona State University ISTB1, 423, East Mall Tempe AZ 85287‐9425 USA; ^2^ Okinawa Institute of Science & Technology Graduate University 1919–1 Tancha Onna‐son Okinawa 904–0495 Japan; ^3^ Faculty of Forestry and Wood Sciences Czech University of Life Sciences Kamycka 129, 16521 Praha Czech Republic

**Keywords:** collective behavior, evolutionary convergence, nest construction, parallel evolution, parameter tuning, self‐organization

## Abstract

Animal collective behaviors give rise to various spatial patterns, such as the nests of social insects. These structures are built by individuals following a simple set of rules, slightly varying within and among species, to produce a large diversity of shapes. However, little is known about the origin and evolution of the behavioral mechanisms regulating nest structures. In this study, we discuss the perspective of inferring the evolution of collective behaviors behind pattern formations using a phylogenetic framework. We review the collective behaviors that can be described by a single set of behavioral rules, and for which variations of the environmental and behavioral parameter values produce diverse patterns. We propose that this mechanism could be at the origin of the pattern diversity observed among related species, and that, when they are placed in the proper conditions, species have the behavioral potential to form patterns observed in related species. The comparative analysis of shelter tube construction by lower termites is consistent with this hypothesis. Although the use of shelter tubes in natural conditions is variable among species, most modern species have the potential to build them, suggesting that the behavioral rules for shelter tube construction evolved once in the common ancestor of modern termites. Our study emphasizes that comparative studies of behavioral rules have the potential to shed light on the evolution of collective behaviors.

## Graphical abstract

Understanding the origin of complex traits has been one of the primary focuses of evolutionary biologists since Darwin. One such trait is the nest structure of social insects, which is built by individuals following a simple set of behavioral rules. Here, we review the litterature on termite nest constructions in a phylogenetic framework and propose that parameter tuning of a common set of behavioral rules is at the origin of the diversity of nest shapes. Our reconstruction of the evolution of termite shelter tube constructions supports this idea, suggesting that the ability to build shelter tubes, and thus the behavioral rules used by termites to build them, originated once in the common ancestor of modern termites.

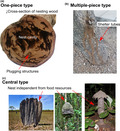

## 1 INTRODUCTION

Many animal species are social and perform collective behaviors. For example, groups of individuals coordinate their movements to create the cohesive motion of shoals, flocks, and insect swarms (Couzin, Krause, James, Ruxton, & Franks, 2002). Collective behavior can also create physical structures, as is the case for nests of social insects (Perna & Theraulaz, 2017). The formation of these patterns is achieved through self‐organization, with collective phenomena emerging from individuals responding to local stimuli without any knowledge of the global pattern (Camazine et al., 2001). One major goal of researchers studying collective behavior is to determine the evolutionary processes leading to complex pattern formations (Bonabeau, 1998; Duarte, Weissing, Pen, & Keller, 2011; Gordon, 2016). Some studies have simulated evolutionary processes using swarms of robots or virtual animals and have revealed that coordinated collective behaviors are adaptive traits under natural selection (Ioannou, Guttal, & Couzin, 2012; Duarte, Pen, Keller, & Weissing, 2012; Fujisawa, Ichinose, & Dobata, 2019). Some studies have also explored the core mechanisms shared across different taxonomic groups (e.g., Schmickl & Karsai, 2018). However, the actual evolutionary history of collective behaviors has rarely been explored, probably because of the challenges and time constraints linked to the systematic acquisition of behavioral data from a significant number of species. Thus, despite the advances in molecular phylogenetics of many animal groups, phylogenetic information remains to be included in studies of self‐organized collective behaviors.

Complex patterns can emerge from simple individual behavioral rules governing interactions among individuals. Models of self‐organized collective behaviors have shown that a limited set of behavioral rules can produce a large diversity of patterns through quantitative modification of parameters such as individual states, environmental conditions, or social conditions (Franks, Gomez, Goss, & Deneubourg, 1991; Karsai & Penzes, 1998; Pratt & Sumpter, 2006). This mechanism, called parameter tuning, potentially explains variations of patterns observed among populations and species living in different environments (Camazine et al., 2001).

**FIGURE 1:**
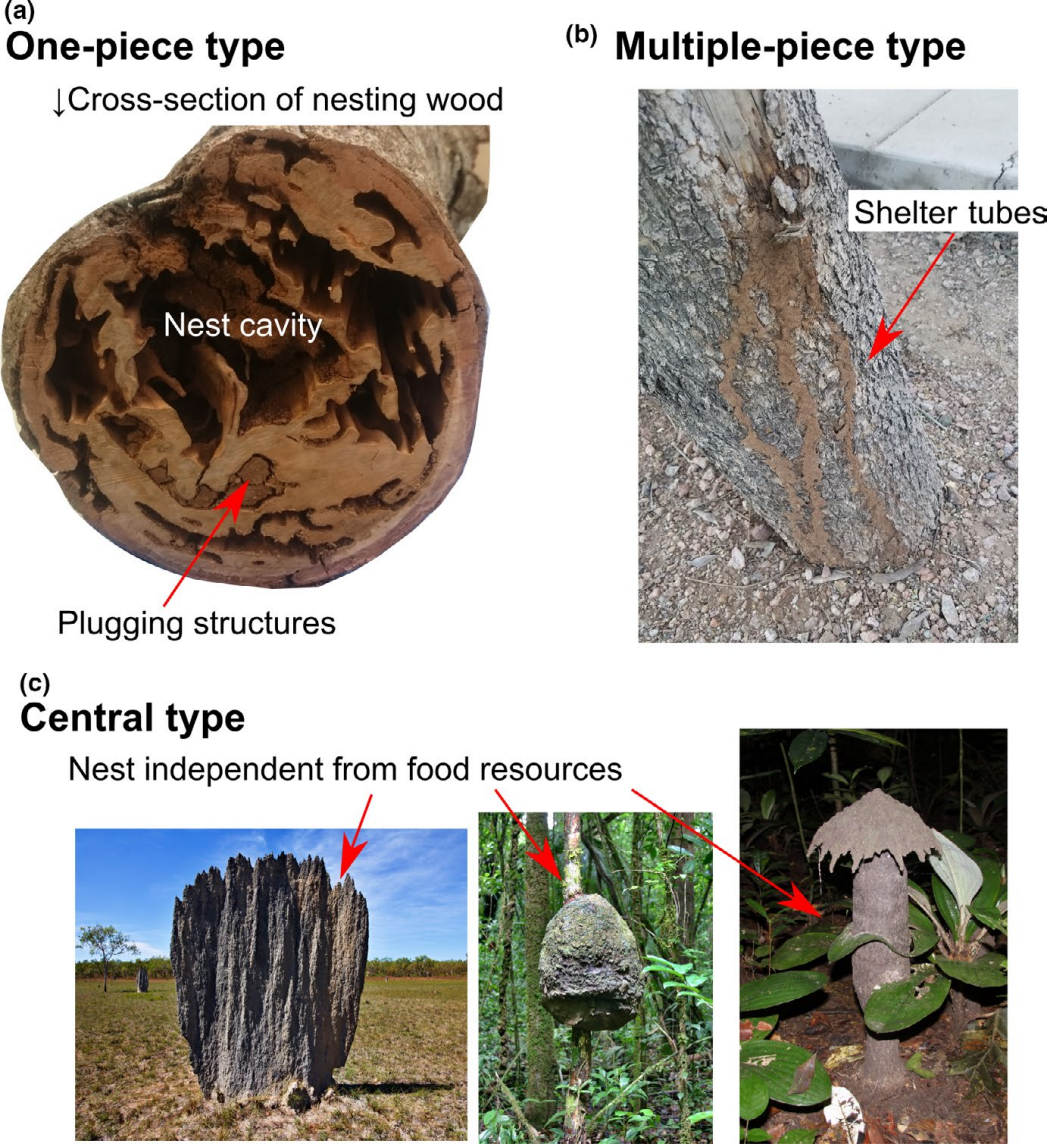
Structures built by termite species adopting the three nesting strategies of Abe (1987). (a) Cross section of nesting wood occupied by the one‐piece nester *Neotermes sugioi*, Kalotermitidae, exposing nest cavities and plugging structures. (b) Shelter tubes built by the multiple‐piece nester *Heterotermes aureus*, Rhinotermitidae, connecting dead parts of the living tree through underground galleries. (c) Nests built by the separate‐piece nesters *Amitermes meridionalis* (left) and *Cubitermes* sp. (right), Termitidae. Separate‐piece nesters build nests with complex internal structures, separated from their food resources. Photographs C are reproduced with the permission of Jan Šobotník

Nests of termites are remarkable spatial patterns formed by the collective behavior of many individuals. Nests present a wide diversity of shapes (Emerson, 1938; Noirot, 1970), ranging from simple barricades built within wood, to networks of shelter tubes connecting multiple nest sites, or complex sponge‐like structures embedded within large mounds (Emerson, 1938) (Figure 1). These structures often have a defensive role, acting as physical barriers, inside nests and during foraging, that protect against predators (Tuma, Eggleton, & Fayle, 2019). All termites are capable of building structures of variable complexity, indicating that the origin of building behavior in termites is ancient, and became more complex more recently in several lineages (Emerson, 1938; Inward, Vogler, & Eggleton, 2007). Here, we hypothesize that parameter tuning of shared behavioral rules could explain the diversity of the structures built by termites. We argue that most species share similar behavioral rules, but species‐specific environmental and physiological states result in species‐specific structures. Should this hypothesis be correct, we expect, among others, that the ability to build shelter tubes is a trait that modern termites inherited from their common ancestor. And thus, we also expect that many termite species have retained this ability, including species that do not build shelter tubes in natural conditions.

In this paper, we show that the behavioral potential for collective building is widely shared across termite species, which arguably facilitated the evolution of diverse structures. We first review the concept of parameter tuning and show that it is commonplace in nature. We then review the literature on termite building behavior and infer its evolution using the latest termite phylogenetic tree. We further reconstruct the evolution of shelter tube construction in termites and provide possible evolutionary scenario inferred from our current understanding of shelter tube building abilities across termite species. Finally, we discuss the limitation of past studies and the promise of studying behavioral phenomena in their broader evolutionary context.

## 2 THE EMERGENCE OF DIVERSE PATTERNS THROUGH SELF‐ORGANIZATION PROCESS

Group‐level patterns, resulting from self‐organized collective behaviors, vary in size and shape in a species‐specific manner (Couzin et al., 2002; Perna & Theraulaz, 2017). Group‐level patterns emerge from interacting individuals following simple behavioral rules. Theoretical studies have shown that the distinct patterns observed within and among species can emerge from quantitative variations of a single set of interaction rules (Bonabeau et al., 1998; Camazine et al., 2001; Franks et al., 1991; Karsai & Penzes, 1998; Khuong, Theraulaz, Jost, & Perna, 2011; Mizumoto, Kobayashi, & Matsuura, 2015; Ocko, Heyde, & Mahadevan, 2019; Theraulaz & Bonabeau, 1995). This parameter tuning mechanism allows for complex patterns to emerge from limited behavioral complexity at the individual level. Parameters can be of two types: behavioral and environmental (Camazine et al., 2001). Behavioral parameters are intrinsic characteristics of each organism (e.g., motion speed), while environmental parameters arise from socio‐environmental conditions (e.g., group size). Empirical studies have shown that parameter tuning within species often follows different socio‐environmental contexts (Deneubourg, Grégoire, & Le Fort, 1990; Pratt & Sumpter, 2006). Here, we review the parameter tuning mechanisms in the context of the collective building behavior of social insects.

During the process of building structures, group members can indirectly interact by changing the spatial distribution of building materials. This type of coordination is called stigmergy (Grassé, 1959) and can result in the formation of various spatial patterns by parameter tuning. For example, in the ant *Messor sanctus*, the spatial pattern of cemeteries results from the behavior of workers reacting to changes of local density of nestmate corpses (Theraulaz et al., 2002). Ants follow one simple behavioral rule: They collect corpses where their density is low and bring them to the “cemetery,” where corpse density is high (Jost et al., 2007; Theraulaz et al., 2002). In this system, behavioral parameters, such as collecting rates or dropping rates, are affected by environmental parameters, such as temperature or speed of airflow, resulting in a distinctly shaped cemetery (Jost et al., 2007). The initial density of corpses also affects the collective outcome without any changes of behavioral responses, and a loose cluster of corpses appears very slowly at low corpse density, while a tight cluster rapidly emerges at high corpse density (Theraulaz et al., 2002). This example illustrates how variations in environmental parameters affect individual responses, changing the way individuals interact, and ultimately modifying building patterns.

The nests of social insects are unique structures formed by the collective building behavior of numerous interacting workers. Termite construction is a classic example of stigmergic interactions among individuals. Construction is initiated by workers randomly placing building materials (e.g., soil pellets) containing the cement pheromones (Bruinsma, 1979). The cement pheromones attract workers to the construction sites, promoting further building and deposition of cement pheromones, thence exerting positive feedback (Grassé, 1959). This system has been mathematically modeled and simulated assuming that workers follow the abovementioned simple set of rules, and it was shown that the quantitative variations of associated parameters are sufficient to give rise to the multitude of nest structures observed in termites (Bonabeau et al., 1998; Mizumoto et al., 2015; Ocko et al., 2019). Local conditions influence the type of structures (e.g., pillars, walls, royal chamber, or shelter tubes) built by termites. For example, the queen presence could initiate the construction of the royal chamber (Bonabeau et al., 1998). Variations of shelter tube patterns among colonies can be explained by colony‐specific sensitivity to cement pheromones (Mizumoto et al., 2015). In the same vein, interspecific variation in the shape of mounds can be described by the tuning of two parameters related to stigmergy and thermodynamics (Ocko et al., 2019).

The variations of nest structures observed in social insects are remarkably well described by species‐specific parameter tuning. Studies combining observations and simulations provide the deepest insight. In the social wasp genus *Polistes*, variations in parameter values of behavioral rules, and variations in colony size, are at the origin of the diversity of nest shapes observed in the genus (Karsai & Penzes, 1998; Karsai & Wenzel, 1998). Another example comes from the ant genus *Lasius*. A simulation model incorporating two behavioral rules, stigmergy and body template, can reproduce the nest of *Lasius niger*, *L. fuliginosus,* and *L. pallitarsis*, only assuming changes in the evaporation rates of pheromones (Khuong et al., 2011). Unfortunately, many of the above studies are mainly based on simulations and rely upon untested assumptions. Thus, further empirical studies are required to support the modeling work of collective building in social insects. For example, the use of cement pheromones to guide construction in termites has been questioned by several empirical observations (Bardunias & Su, 2010; Fouquet, Costa‐Leonardo, Fournier, Blanco, & Jost, 2014; Green, Bardunias, Turner, Nagpal, & Werfel, 2017; Petersen et al., 2015). In mound‐building termites, stigmergic interaction could be mediated by nonpheromonal mechanisms, including environmental heterogeneity, physical properties, and moisture (Carey et al., 2019; Fouquet et al., 2014). Alternatively, a recent model combining excavation and deposition described the early stage of construction more accurately than stigmergy (Green et al., 2017). This model might be a good candidate to explain interspecific variation. Therefore, detailed observations on a representative set of species are required to validate parameter tuning as an explanation for variations of nest structures.

## 3 CLASSIFICATION OF TERMITE LIFE TYPES

A first step to understand the evolutionary processes of collective behaviors is to classify the patterns observable in nature. Termite nests are remarkable examples of spatial patterns and are built through a combination of excavation and construction, both of which play essential roles in nest formation (Lee, Su, Song, & Lee, 2020; Green et al., 2017). In this study, we focus on the structures built by active construction because, in many primitive species, excavation is often the result of feeding activities and is difficult to distinguish from nest building (Emerson, 1938). Termites construct a wide array of structures, varying in size and shape among species. There have been several attempts to classify the nesting behavior of termites (Abe, 1987; Emerson, 1938; Korb, 2008; Lee & Wood, 1971). Among these, that of Abe (1987) is the most comprehensive and widely used. Abe recognizes three categories of nest developments: single‐piece nesters (one‐piece type), multiple‐piece nesters (intermediate type), and separate‐piece nesters (central type). Note that these categories are not entirely distinctive, but rather lie on a continuum with intermediate types between them.

**FIGURE 2:**
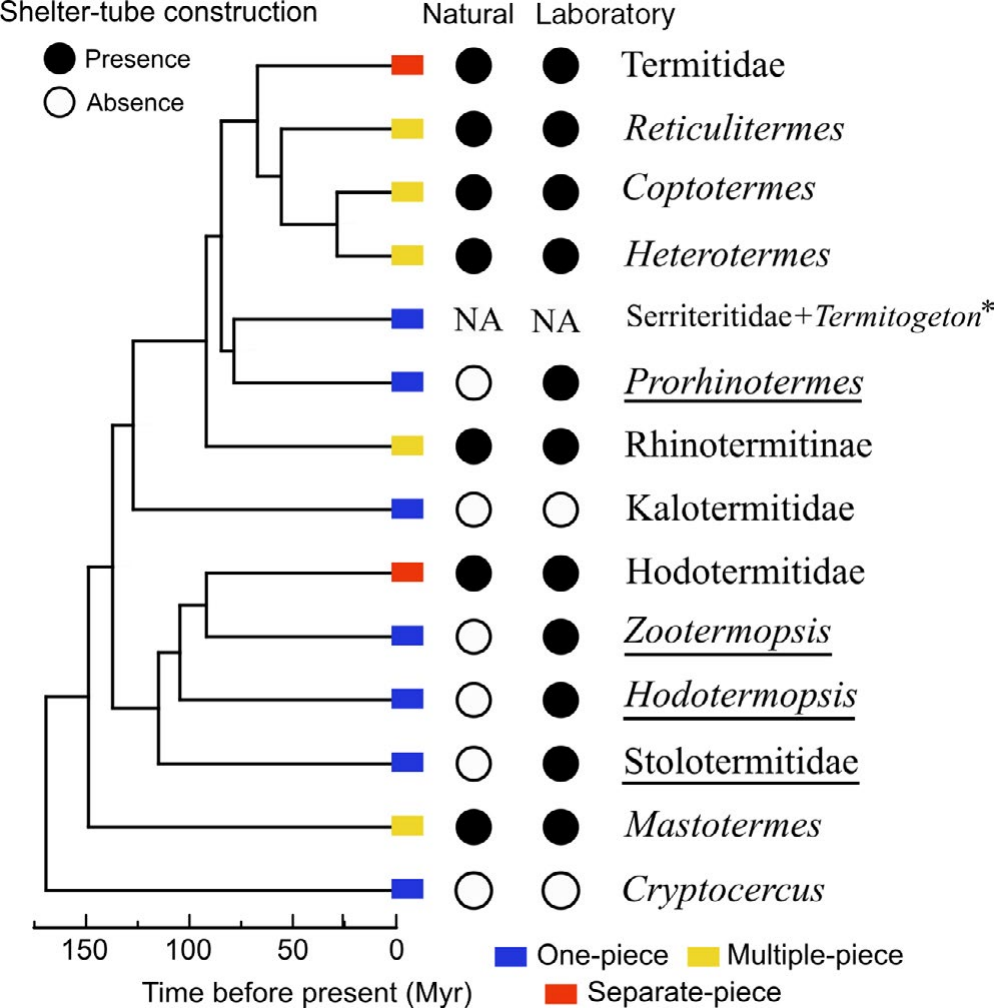
Phylogenetic tree of termites, simplified from Bourguignon et al. (2015), showing the widely shared ability to build shelter tubes in termites. The genera retaining the ability to build shelter tubes without using them in natural conditions are underlined. Some genera were lumped together into higher‐ranked monophyletic lineages to account for the lack of research. The shelter tube building ability of Stolotermitidae is based on observations on *Stolotermes*, and that of Hodotermitidae is based on observations on *Anacanthotermes*. Species of Kalotermitidae were lumped together as they all lack the ability for shelter tube construction. We found no information about shelter tube construction in Serritermitidae + Termitogeton

One‐piece nesters include species with nests consisting of a single piece of wood (including damp wood, dry wood, and dead branches on living trees), serving both as shelter and food source. These species construct simple structures within their wood‐piece nests, such as separate cells within the excavated wood, and barricades to plug cell openings (Figure 1a). One‐piece nesters include Archotermopsidae, to which belong *Hodotermopsis* and *Zootermopsis*, all species of Stolotermitidae, Stylotermitidae, and Serritermitidae, almost all species of Kalotermitidae, and some species of Rhinotermitidae, such as *Prorhinotermes* and *Termitogeton* (Figure 2). Note that some species, classified as single‐piece nesters, occasionally move out of their wood‐piece nest and colonize neighboring wood items (Bourguignon, Chisholm, & Evans, 2016; Rupf & Roisin, 2008). This nesting lifestyle is often considered primitive because it resembles that of the termite sister group, *Cryptocercus*, which excavate wood without constructing external structures (Emerson, 1938).

Multiple‐piece nesters include species forming colonies encompassing multiple pieces of wood. Similarly to one‐piece nesters, multiple‐piece nesters excavate their nests directly within wood pieces. In addition, to ensure safe traveling among wood pieces, multiple‐piece nesters often excavate belowground tunnels or construct aboveground shelter tubes resembling pipes made of wood pieces, soil, and termite excretions (Figure 1b). Termites generally build shelter tubes against substrates, in which case shelter tubes are composed of tube sections (Mizumoto & Matsuura, 2013). Multiple‐piece nesters include *Mastotermes darwinensis* (Mastotermitidae), *Paraneotermes simplicicornis* (Kalotermitidae), most species of Rhinotermitidae, and some species of Termitidae (Abe, 1987) (Figure 2).

Separate‐piece nesters include species building nests physically separated from their food sources. Nests can be subterranean, built at the soil surface in the shape of large mounds, or arboreal (Figure 1c). Nests of separate‐piece nesters display a large variety of forms, both in external appearance and internal structures. Highly complex internal structures mainly consist of chambers and interconnected corridors (Perna & Theraulaz, 2017), and of more specialized structures such as the royal chamber, fungus gardens, and chimney. The separate‐piece nesters include all Hodotermitidae, some species of Rhinotermitidae, and most species of Termitidae (Figure 2).

The nesting life type of the most recent ancestor of modern termites is still unknown. The separate‐piece nesting life type, and the construction of complex nests to which it is associated, are clearly derived traits (Inward et al., 2007). Similarly, given that shelter tube construction is absent in cockroaches (Bell, Roth, & Nalepa, 2007), the most recent common ancestor of termites and *Cryptocercus* was certainly a one‐piece nester unable to build shelter tubes. However, whether the most recent common ancestor of modern termites was one‐piece nester or multiple‐piece nester is still a subject of debate (Bourguignon et al., 2016; Watson & Sewell, 1985). The traditional view of termite evolution posits that one‐piece nesting is ancestral (Abe, 1987; Inward et al., 2007). Following this scenario, the ancestor of modern termites lived in small colonies, composed of a queen, a king, and their offspring, all cloistered in their wood‐piece nest (Thorne, 1997). This view is intuitive, as it suggests a gradual increase from simplicity toward complexity of social structure. However, there is a possibility that the most recent common ancestor of modern termites already went through the transition from one‐piece nesters to multiple‐piece nesters, as has been suggested by some studies (Bourguignon et al., 2016; Watson & Sewell, 1985). In support of this theory, *Mastotermes darwiniensis*, the sister group of all other modern termites, is a multiple‐piece nester, and many basal termite species can move out of their wood‐piece nest and colonize new food sources (Bourguignon et al., 2016).

In this paper, with the idea of parameter tuning of self‐organization in mind, we hypothesize that the different structures built by one‐piece nesters and multiple‐piece nesters are produced by the same set of behavioral rules, with different values for behavioral and environmental parameters. In other words, we hypothesize that one‐piece nesters are able to build the structures typically attributed to multiple‐piece nesters. The hypothesis implies that the most recent common ancestor of modern termites, should it be one‐piece nester or multiple‐piece nester, was already able to build complex structures. This hypothesis can be tested by examination of the building abilities of a set of termite species sampled across the termite phylogeny.

## 4 EVOLUTION OF SHELTER TUBE CONSTRUCTION IN TERMITES

Important progress has been made in phylogenetics since Emerson (1938) first studied the evolution of termite constructions in a phylogenetic framework. In this paper, using the latest molecular phylogenetic tree, we inferred the evolutionary processes that led to the diversity of structures built by modern termites. The hypothesis implies that all species share the same behavioral rules and have the potential to construct basic elements of complex structures. We examined the evolutionary patterns of shelter tube constructions in termites, especially focusing on the transition between one‐piece nesters and multiple‐piece nesters. Shelter tubes are easily distinguishable from other building structures, and their presence has been largely reported in the literature. As the primary use of shelter tubes is to connect separate nesting and foraging sites (wood pieces), one‐piece nesters are not expected to use them. However, the fact that they do not use shelter tubes in natural condition does not necessarily imply they lack the ability to build them.

We did not consider belowground tunnels, which have the same function as shelter tubes and may experience similar evolutionary processes (Mizumoto, Bardunias, & Pratt, 2019). Yet, these two structures can be distinguished in terms of their complexity; tunneling is mainly performed by excavation and can be considered as an extension of feeding activities (Emerson, 1938), while shelter tube construction requires specific deposition patterns to form tube‐like structures. We collected literature data to shed light on the evolution of shelter tube building abilities in termites. We focused our endeavors on the 150‐million‐year‐old lower termites, which form a paraphyletic group including all termites but Termitidae, as they can provide insight into the origin of termite social systems (Abe, 1987; Inward et al., 2007). We did not further consider the 50‐million‐year‐old higher termites (all Termitidae) in this paper.

We used Google Scholar to collect literature data on the ability of termites to build shelter tubes. We used the following combination of keywords: shelter tube, runway, covered trail, shelter gallery, mud tube, and covered pathway. We repeated the search for every genus of lower termites. We also tracked down the original references cited by every relevant paper we found. Also, we searched on google for images and videos showing shelter tube construction. We compiled literature information for all termite lineages and inferred the evolution of shelter tube building abilities in termites, using the molecular phylogeny of Bourguignon et al. (2015). This study, based on 66 mitochondrial genomes, provides a robust phylogenetic tree, largely consistent with the transcriptome‐based phylogenetic tree of Bucek et al., 2019, and including all major clades of lower termites.

We found that at least one species was reported to build shelter tubes in seven of the nine extant families of termites. Multiple‐piece nesting strategy, and thus shelter tube construction in a natural condition, are believed to have several independent origins in termites (Inward et al., 2007). However, laboratory observations indicate that most modern termite species have the potential to build shelter tubes, which suggests a single origin of shelter tubes in the common ancestor of all termites (Table 1 and Figure 2). We used the ace function implemented in R package phytools (Revell, 2012) to carry out a preliminary ancestral state reconstruction of shelter tube building abilities in termites. We used a maximum likelihood model with an equal rate of transition among states and the tree presented in Figure 2, assuming Serritermitidae + *Termitogeton* are unable to build shelter tubes. In this analysis, a single origin of shelter tube construction in the ancestor of modern termites is supported with a probability of 95.6%. Under this scenario, the ability to build shelter tubes was lost at least once in Kalotermitidae. Among the species that use shelter tubes in natural conditions, some species of Rhinotermitidae, such as *Reticulitermes* and *Coptotermes*, build extensive shelter tube networks, while the use of shelter tubes is occasional in Mastotermitidae (*Mastotermes darwiniensis*;Emerson, 1938; Lee & Wood, 1971) and Hodotermitidae (*Anacanthotermes turkestanicus*; Khamraev et al., 2008). Interestingly, several species classified as one‐piece nesters retained the ability to build shelter tubes, including species in Archotermopsidae (*Hodotermopsis sjostedti* and *Zootermopsis nevadensis*; Bordereau & Pasteels, 2011, Figure 3), Stolotermitidae (*Stolotermes ruficeps*; Morgan, 1959), and Rhinotermitidae (*Prorhinotermes inopinatus*; Rupf & Roisin, 2008).

**TABLE 1:** Detailed information about shelter tube construction in lower termites

Family	Genus	Classification	Site	References	Image
Mastotermitidae	*Mastotermes*	Multiple‐piece	Fields	Emerson (1938), Lee and Wood (1971)	Video from youtube: https://www.youtube.com/watch?v=uV2YmJrxEc0, by Tactic Pest Control, 2018, last checked on 3/29/2020
Stolotermitidae	*Stolotermes*	One‐piece	Laboratory	Morgan (1959)	Drawing (Morgan, 1959)
Archotermopsidae	*Hodotermopsis*	One‐piece	Laboratory	Bordereau and Pasteels (2011)	Picture (Bordereau & Pasteels, 2011) (Figure 3)
	*Zootermopsis*	One‐piece	Laboratory	Bordereau and Pasteels (2011)	Picture (Figure 3)
Hodotermitidae	*Anacanthotermes*	Separate	Field	Khamraev et al. (2008)	Picture^*1^ (Khamraev et al., 2008)
Rhinotermitidae	*Schedorhinotermes*	Multiple‐piece	Field	Lee and Wood (1971)	Picture^*2^ (Lee & Wood, 1971)
	*Prorhinotermes*	One‐piece	Laboratory	Rupf and Roisin (2008)	Picture (Rupf & Roisin, 2008)
	*Heterotermes*	Multiple‐piece	Field	Shelter tube constructions are very common in these genera (e.g., Mizumoto et al., 2015for the video of *Reticulitermes* and Figure 1b for *Heterotermes*)	
	*Coptotermes*	Multiple‐piece	Field		
	*Reticulitermes*	Multiple‐piece	Field		
Potentially similar behavior with shelter tube construction					
Kalotermitidae	*Neotermes*	One‐piece	Field^*3^	Waterhouse and Norris (1993)	Picture (Waterhouse & Norris, 1993)

Note

Some are not true shelter tube construction, where *1 indicates mud‐tube coating grass, *2 indicates soil sheeting, and *3 indicate a net‐like pattern of grooves covered with construction.

**FIGURE 3:**
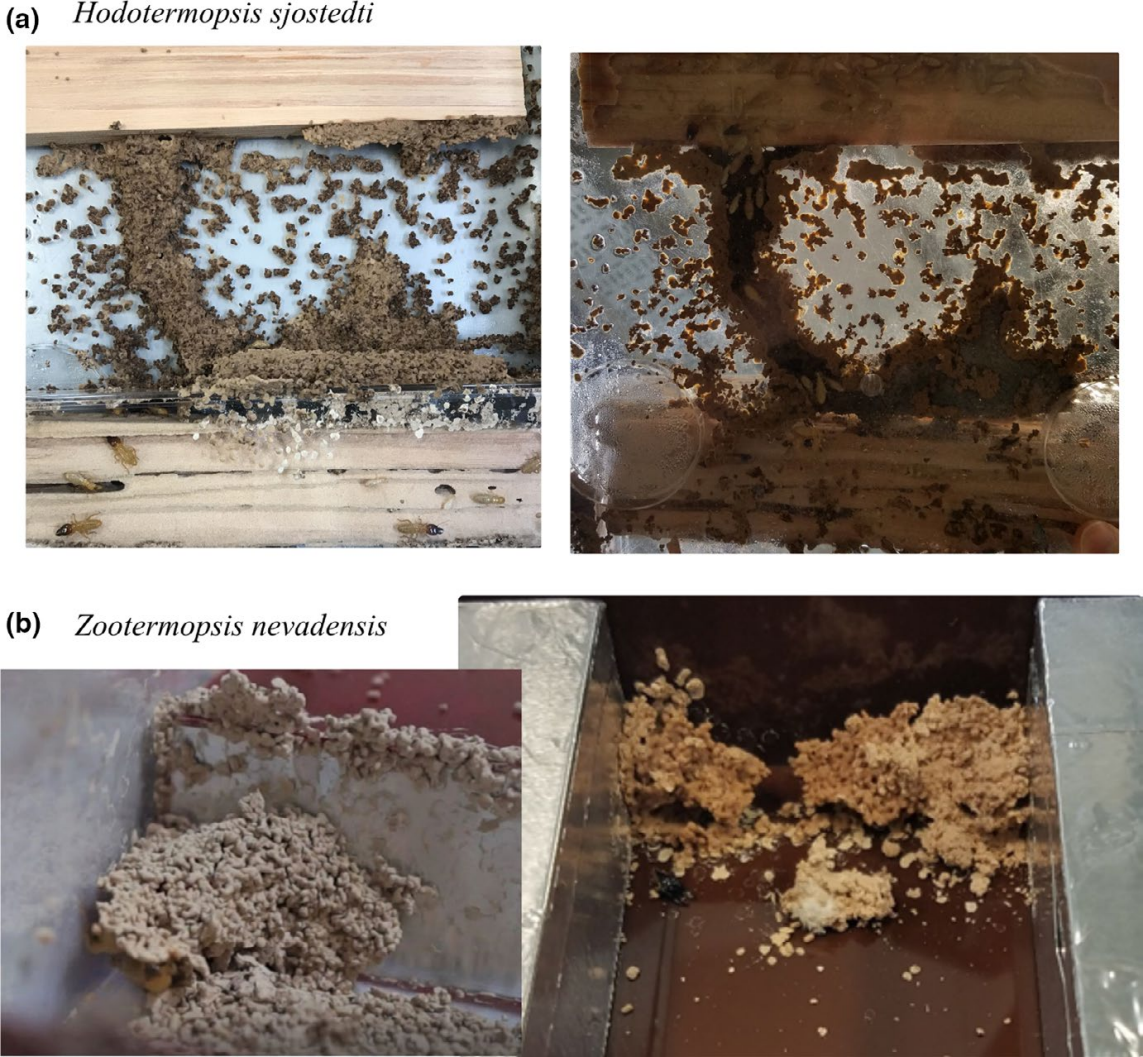
Pictures showing shelter tubes constructed by (a) *Hodotermopsis sjostedti* and (b) *Zootermopsis nevadensis*. Observations were made under laboratory conditions. The shelter tube construction was stimulated by starving condition and nest damage

Parameter tuning provides a framework to explain the variation of shelter tube construction in termites. Assuming that the ability of modern termites to build shelter tubes was inherited from their common ancestor, all modern termite species may share certain behavioral rules for construction. These inherited behavioral rules allow many species to build shelter tubes in natural conditions, but they are also used to build other structures, such as partitions and barricades. For some species, the parameter values of the behavioral rules are out of range for shelter tube construction in natural conditions, thence these species build no shelter tubes in natural conditions and use the behavioral rules for other construction purposes (Mizumoto, 2018). However, the unnatural conditions experienced in laboratory could alter parameter values, allowing a resurgence of shelter tube construction in species that usually do not build them. The construction of these laboratory‐built shelter tubes tends to be slower and unstable compared to that of species building shelter tubes in nature (Figure 3), suggesting that the tuning of behavioral parameters also plays an important role for the evolution of shelter tube constructions.

The collective building behavior of several species of Kalotermitidae has been relatively wells tudied, and none were found able to build shelter tubes. However, it is not excluded that some species, especially those diverging early on in the Kalotermitidae tree of life, retain the potential to build shelter tubes. One example is *Paraneotermes simplicicornis*, the only definite multiple‐piece nesting species in the Kalotermitidae, which can dig galleries through the soil (Abe, 1987; Light, 1937; Mizumoto, Bardunias, et al., 2019). In addition, some populations of *Neotermes rainbowi* are known to be multiple‐piece nesters, and construct tunnel‐like structures made of bark chips on the surface of palm tree trunks; however, this behavior is absent in other species of *Neotermes* (Waterhouse & Norris, 1993). Although actual shelter tubes have never been observed in Kalotermitidae, more studies on the behavioral rules of Kalotermitidae are required to build a reliable model of the evolution of collective building behavior in termites.

## 5 CONCLUSION AND PERSPECTIVES

In this paper, we suggest that modern termites inherited their ability for shelter tube construction from their common ancestor and that most species have retained the potential since then. The ability to build shelter tubes is shared by most modern termite species, including some one‐piece nesters that do not use shelter tubes in natural conditions (Figure 2). One notable exception is the Kalotermitidae, whose behavioral potential for shelter tube constructions was either secondarily lost, or extremely altered. We argue that the potential for shelter tube construction is maintained in most termites because most termites share the same behavioral rules, the tuning of which may have facilitated the repeated evolution of similar nest structures across different termite lineages. This process is analogous to genetic accommodation, which posits that independent changes in gene expression in population/species sharing genotypes can lead to parallel evolution of specific phenotype (Rajakumar et al., 2012).

With the idea of parameter tuning in mind, we propose that species building no shelter tubes in natural conditions conserve the potential to do so by using the behavioral rules they use to construct other types of structures, such as partitions and barricades. These simple structures are present across all termite species, including in most Kalotermitidae (Emerson, 1938; Wilkinson, 1962). For example, colony founders of *Incisitermes minor* seal the entrance of royal chamber with barricade made of cement feces (Himmi et al., 2014). The behavioral mechanism for barricade construction might be homologous to that of shelter tube construction. Past observations found that *Zootermopsis nevadensis* builds barricades in laboratory using a positive feedback process (Mizumoto, 2018), which is also used by termites during shelter tube construction (Bonabeau et al., 1998; Mizumoto et al., 2015). More studies are needed to determine the species‐specific parameter values taken by behavioral rules for the construction of shelter tubes, and other structures, across a representative set of termite species.

What are the crucial factors influencing the construction of shelter tubes in one‐piece nesters? Firstly, as the primary function of shelter tubes is to connect multiple wood pieces, their construction implies the need for movements between the original nest and new wood pieces. Conditions such as starvation or nest damage can stimulate nest emigration and shelter tubeconstruction (Rupf & Roisin, 2008) (Figure 3), possibly through changes of behavioral parameters, such as the location of material deposition. Note that these conditions can also stimulate alate differentiation and dispersal of colony members, especially in species in which the work force is composed of totipotent pseudergates (Korb & Lenz, 2004). Secondly, the environmental parameters also need to be tuned to facilitate shelter tube construction. For example, *Stolotermes ruficeps* does not build shelter tubes in natural conditions, but creates them in laboratory under highly humid conditions (Morgan, 1959). Finally, the change of building materials might be important in the evolution of shelter tube construction. Multiple‐piece or separate‐piece nesters, that build shelter tubes in natural conditions, use specifically soils and wood carton for construction (Emerson, 1938; Oberst, Lai, & Evans, 2016; Zachariah, Das, Murthy, & Borges, 2017), while one‐piece nesters use random materials for construction, including wood frass, solid feces and any soil or dust that may be available (Mizumoto, 2018; Morgan, 1959). This is associated with changes of both environmental parameters (availability of materials) and behavioral parameters (preference of materials).

Like shelter tubes, underground tunnels allow safe travel among different wood pieces. And, as is the case for shelter tubes, several basal one‐piece nesting termites dig tunnels in the soil under laboratory conditions (Castle, 1934; Nkunika, 1988; Waterhouse & Norris, 1993). These observations suggest that the common ancestor of modern termites was a multiple‐piece nester able to build shelter tubes and tunnel through soil. However, in disagreement with this hypothesis, a recent comparative study found that the behavioral rules for collective excavation of tunnels shared by *Reticulitermes tibialis* and *Heterotermes aureus* (Rhinotermitidae) are not shared by *Paraneotermes simplicicornis* (Kalotermitidae) (Mizumoto, Bardunias, et al., 2019), possibly indicating an independent origin of tunneling in *P. simplicicornis* following the secondary loss of shelter tubes in Kalotermitidae. These results indicate that the comparison of a limited set of species is insufficient to determine the evolutionary processes at the origin of tunneling. A detailed comparison of the behavioral rules, determined from a comprehensive set of termite species, is required to draw a general conclusion about the origin of tunneling.

Although our analysis focused on the evolutionary transition between one‐piece and multiple‐piece nesters, the concept of parameter tuning of behavioral rules can be extended to the evolution of more complex nest structures, such as the mounds built by many separate‐piece nesters. Because of the huge gap in the complexity of structures built by one‐piece nesters and separate‐piece nesters, it is improbable that all lower termites, especially one‐piece nesters, have the potential to build entire mounds under particular laboratory condition. However, some lower termites are able to build elements of complex mound structures. For example, many species of *Schedorhinotermes* and *Coptotermes* inhabit wood pieces, that also serve as food sources, within which they construct carton nest structures resembling those of higher termites (Chouvenc, Efstathion, Elliott, & Su, 2013; Kaib, Husseneder, & Epplen, 1996), or a few species even build entire mound (Oberst et al., 2016). We posit that these species already had a set of behavioral rules and that parameter tuning of these rules facilitated the evolution of mound‐building.

Mound‐building has also multiple origins among higher termites, as exemplified by the Australian Nasutitermitinae in which it evolved independently in at least six lineages (Arab et al., 2017). As is the case for shelter tube construction, there is a possibility that all species of Nasutitermitinae share the same behavioral rules and that the species that do not build mounds under natural conditions are able to do so under unusual conditions. In this scenario, parameter tuning of ecological and behavioral parameters facilitate the repeated evolution of mound‐building in termites. More data on behavioral mechanisms are required to understand the evolutionary process of termite construction.

As Darwin first argued, complex adaptive traits can evolve gradually. By decomposing complex traits into the multiple components they are made of, their gradual evolution can be tracked on phylogenetic trees (Suzuki, 2017). Collective behaviors of group‐living animals are complex adaptive traits, which can be decomposed into behavioral rules with environmental and behavioral parameters, and studied in a phylogenetic framework. For example, it was shown that swarming patterns formed by locusts at high density evolved multiple times from solitary grasshoppers (Cullen et al., 2017), and nonswarming relatives also exhibit density‐dependent behaviors in laboratory conditions (Sword, 2003). Another example is that of fish schooling behavior, which was lost and regained repeatedly (Kasumyan & Pavlov, 2018), with many species, even including extinct species, having similar repulsion and attraction behavioral rules for coordinated collective motion (Herbert‐Read et al., 2011; Mizumoto, Miyata, & Pratt, 2019). These examples illustrate how the evolution of collective behaviors can be studied in a phylogenetic framework. We hope that this paper encourages further empirical studies using the concept of parameter tuning, coupled with molecular phylogenies, to study the evolution of self‐organizing systems.

## CONFLICT OF INTEREST

The authors declare no competing interests.

## AUTHOR CONTRIBUTION

**Nobuaki Mizumoto:** Conceptualization (lead); Data curation (lead); Investigation (equal); Visualization (equal); Writing‐original draft (equal); Writing‐review & editing (equal). **Thomas Bourguignon:** Formal analysis (lead); Visualization (equal); Writing‐original draft (equal); Writing‐review & editing (equal).

## Data Availability

All data for producing Figure 2 will be deposited in Dryad (https://doi.org/10.5061/dryad.bg79cnp7q).
